# Exploring the Ligand-Protein Networks in Traditional Chinese Medicine: Current Databases, Methods, and Applications

**DOI:** 10.1155/2013/806072

**Published:** 2013-06-02

**Authors:** Mingzhu Zhao, Qiang Zhou, Wanghao Ma, Dong-Qing Wei

**Affiliations:** National Key Laboratory of Microbial Metabolism and College of Life Sciences and Biotechnology, Shanghai Jiao Tong University, 800 Dongchuan Road, Minhang District, Shanghai 200240, China

## Abstract

The traditional Chinese medicine (TCM), which has thousands of years of clinical application among China and other Asian countries, is the pioneer of the “multicomponent-multitarget” and network pharmacology. Although there is no doubt of the efficacy, it is difficult to elucidate convincing underlying mechanism of TCM due to its complex composition and unclear pharmacology. The use of ligand-protein networks has been gaining significant value in the history of drug discovery while its application in TCM is still in its early stage. This paper firstly surveys TCM databases for virtual screening that have been greatly expanded in size and data diversity in recent years. On that basis, different screening methods and strategies for identifying active ingredients and targets of TCM are outlined based on the amount of network information available, both on sides of ligand bioactivity and the protein structures. Furthermore, applications of successful *in silico* target identification attempts are discussed in detail along with experiments in exploring the ligand-protein networks of TCM. Finally, it will be concluded that the prospective application of ligand-protein networks can be used not only to predict protein targets of a small molecule, but also to explore the mode of action of TCM.

## 1. Introduction

Drug discovery was once an empirical process when the effect of the medicine was purely based on phenotype readout, while the mode of action of drug molecules remained unknown. Later, reductionists began to research on the molecular mechanism of the drug-target interactions, believing that the drug is like a magic bullet towards the functioning targets [1]. This means that a drug takes action on the disease by interacting with one specific therapeutic target. The idea of each drug being like a key (or ligand) matching each “lock” (or protein) has guided the modern drug discovery practice for the last several decades. However, in the recent years, more and more evidence has shown that many drugs exert their activities by modulating multitargets [2–4]. Besides, some drugs interact with antitargets and induce strong side effects [5, 6]. Therefore, it is inappropriate to stick to the paradigm that drug interacts with only one target. How to modulate a set of targets to achieve efficacy while avoiding others to reduce the risk of side effects remains a central challenging task for pharmaceutical industry.

The traditional Chinese medicine (TCM), which has been widely used in China as well as in other Asian countries for a long history, is considered to be the pioneer of the “multicomponent-multitarget” pharmacology [7, 8]. Thousands of years of clinical practices in TCM have accumulated a considerable number of formulae that exhibit reliable *in vivo* efficacy and safety. Based on the methodology of holism, hundreds of different components in a TCM prescription can cure the diseases or relieve the patients by modulating a serial of potential therapeutic targets [9].

In recent years, great efforts have been made on modernization of TCM, most on identification of effective ingredients, and ligands in TCM formulae and functioning targets [10, 11]. Several databases of TCM formulae, ingredients and compounds with chemical structures have been established such as traditional Chinese medicine database (TCMD) [12]. However, the molecular mechanisms responsible for their therapeutic effectiveness are still unclear. On one hand, experimental validation of new drug-target interactions still remains very limiting and expensive, and very few new drugs and targets are identified as clinical applications every year [13, 14]. On the other hand, the complex composition and polypharmacology of TCM make it even harder to conduct a full set of experiments between compounds and targets and elucidate the multitarget mode of action from the holistic view on the biological network level. 

On the contrary, *in silico *methods can predict a large number of new drug-target interactions, construct the drug-target networks, and explore the functional mechanism underlying the multicomponent drug combinations at the molecular level. In the present stage, there have already been successful applications in interpreting the action mechanism of TCM from the perspective of drug-target networks, although the quantity is limited. Compared with the huge amounts of TCM formulae and components, only a small portion of drug-target pairs has been validated by the laborious and costly biochemical experiments. This motivates the needs for constructing models that could predict genuine interacting pairs between ligands and targets, based on the existing small number of known ligand-target bindings.

In this paper, we firstly investigate TCM databases for *in silico* methods that have been greatly expanded in size and data diversity in recent years. On that basis, different screening methods and strategies for identifying active ingredients and targets of TCM are outlined based on the amount of information available, both on sides of ligand bioactivity and the protein structures. Finally, successful applications in this area have been summarized and reviewed, including experimental and computational examples. Learning from the methods in modern western medicine (WM), different computing models and strategies can be used to confirm the effective components and related targets in TCM in order to build the ligand-target networks. One of the research directions of the modernization of TCM is to clarify the mode of action of TCM based on ligand-protein networks. 

## 2. Databases for TCM

Data availability is the first consideration before any virtual screening or data-mining task could be undertaken. The TCM databases can be classified in accordance with several categories, namely, formulae, herbs, and compounds. The formula of TCM is a combination of herbs for treating a disease, while compounds are the bioactive molecules within herbs. In this section, we have summarized a list of databases for TCM herbs, formulations, and compounds, as shown in Table 1.

The elementary units of TCM databases are compounds, the bioactive components that exert efficacy through binding to therapeutic targets. Most of the compounds in TCM databases have two-dimensional structure, while some of them have three-dimensional structures deduced by force field. In most TCM databases, the information of both herbs and compounds are collected while some even have formulae information as well.

The traditional Chinese medicine database (TCMD) contains 23,033 chemical constituents and over 6760 herbs that mainly come from Yan et al. [12]. The query keywords for the database include molecular formula, substructure, botanical identity, CAS number, pharmacological activity, and traditional indications. Only a small proportion of herbs in TCMD have full coverage of compounds while most have partial coverage. Chinese herb constituents database (CHCD) contains information on 8264 compounds derived from 240 commonly used herbs with both botanical and Chinese pinyin names, the part of the herbs that contain the compounds, pharmacological and toxicological information, and other useful information [15]. Qiao et al. [16] have developed 3D structural database of biochemical components which covers 10,564 constituents from 2073 herbs with 3D structures built and optimized using the MMFF94 force field [23]. This database uses MySQL as the data engine and contains detailed information such as basic molecular properties, optimized 3D structures, herb origin, and clinical effects. The TCM database@Taiwan was reported to be the world's largest traditional Chinese medicine database. The web-based database contains more than 20,000 pure compounds isolated from 453 TCM herbs [17]. Both simple and advanced query methods are acceptable in terms of molecular properties, substructures, TCM ingredients, and TCM classifications.

In addition to herbs and compounds, traditional Chinese medicine information database (TCM-ID) [18], TCM drugs information system [19], and comprehensive herbal medicine information system for cancer (CHMIS-C) [20] also collect the information of TCM formulae. TCM-ID is developed by Zhejiang University together with National University of Singapore on all aspects of TCM herbs. TCM-ID currently takes in 1197 TCM formulae, 1313 herbs, and around 9000 compounds. It covers ~4000 disease conditions, and more than half of the compounds have valid 3D structures. The data are collected from creditable TCM books as well as journals, and the records can be retried by different sets of query keywords. TCM drugs information system based on networks of five large databases has also been developed [19]. It includes information of 1712 formulae, 2738 herbs, 16,500 compounds, and 868 dietotherapy prescriptions from the integration of Chinese herb database, Chinese patent medicine database, effective components database of Chinese herbs, Chinese medical dietotherapy prescription database, and Chinese medical recipe database. Herbal medicine information system for cancer (CHMIS-C) integrates the information of 203 formulae that are commonly used to treat cancer clinically as well as 900 herbs and 8500 compounds. The compounds in this database are linked to the entries in National Cancer Institute's database and drugs approved by the U.S. Food and Drug Administration.

The China natural products database (CNPD) [21], marine natural products database (MNPD) [22], and bioactive plant compounds database (BPCD) [15] only focus on the structures of the compounds in TCM and do not contain pertinent information on formulae and herbs. CNPD is built to meet the needs for drug discovery using natural products including TCM and collects the 2D and 3D structures of more than 45,055 compounds. MNPD has a collection of 8078 compounds from 10,000 marine natural products, of which 3200 have bioactivity data. BPCD contains information on 2794 active compounds against 78 molecular targets, as well as the subunits of the target structures to which the compounds bind.

There are other databases on the internet focusing only on the clinical efficacy or side effects of formulae and herbs, without details of compounds. acupuncture.com.au collects the TCM formulae according to their clinical action and efficacy. Both the English and Chinese names of TCM herbs are recorded to facilitate studies using both traditional and modern methods. The dictionary of Chinese herbs contains information on both clinical usage and side effects of the TCM herbs. It also includes the samples of TCM formulae for treating diseases such as cancer, dengue fever, diabetes, and hepatitis B. Besides, the compatibility of TCM herbs and certain drugs is listed to provide biochemical explanation for drug designers. The plants for a future database allows querying of herbs with special medicinal usage and also lists the potential side effects, medical usage, and physical characteristics.

## 3. *In Silico* Methods for Ligand-Protein Interactions

The computational methods for drug discovery based on ligand-protein networks have been increasingly developed and applied in the area of TCM and other drugs in recent years [7, 8]. These methods mainly fall into the territories of ligand-based approach, target-based approach, and machine learning. 

### 3.1. Ligand-Based Approach

The ligand-based approach, also known as the chemical approach, is to reorganize pharmacological characteristics and protein associations, by means of ligand similarities rather than genomic space such as sequence, structural or pathway information. The basic assumption for ligand-based approach is that, regardless that similar chemical structures may interact with proteins in different ways, similar ligands tend to bind to similar targets more than not [24]. The general practice of ligand-based approach is to describe ligands with chemical descriptors, and calculate the similarity coefficient (most commonly, Tanimoto coefficient or *T*_*C*_ [25–27]) between ligands. With the ligand-based descriptions of a protein, one can predict which targets are likely to be hit by a ligand, given its known structure.

In the area of ligand-based virtual screening, researchers have tried to evaluate whether novel ligand-target pairs could be identified, based on the chemical knowledge of ligands and ligand-target interactions. G protein-coupled receptors (GPCRs) are a family of effective drug targets with significant therapeutic value. Many researchers have built support vector machine (SVM) models as well as substructural analysis to describe GPCRs from the perspective of ligand chemogenomics [28]. In particular, the deorphanization of receptors without known ligands was employed using the ligands of the related receptors. For 93% of the orphan receptors, the prediction results are better than random, while for 35% the performance was good. 

A powerful ligand-based prediction method based on features of protein ligands is the similarity ensemble approach (SEA), which was originally used to investigate protein similarity based on chemical similarity between their ligand sets with the main idea that similar ligands might tend to share same targets [3]. SEA calculates *Z*-score and *E*-value by summing up the *T*_*C*_ over a threshold between two ligand sets as indicators to evaluate the possible interaction between two ligand sets in a way similar to BLAST. The similarity threshold for *T*_*C*_ is chosen in a way that the *Z*-score best observes the extreme value distribution (EVD). This method was then applied to predict new molecular targets for known drugs [29]. The author investigated 3000 FDA-approved drugs against hundreds of targets and found 23 new cases of drug-target interactions. By *in vitro* experiments, five of them were validated to be positive with affinities less than 100 nM. Besides Keiser's research, SEA was also used to investigate the off-target effect of some commercial available drugs against the target protein farnesyltransferase (PFTase) [30], and two drugs loratadine and miconazole were found to be able to bind to PFTase. 

The pharmacophore model is perhaps the most widely used methods that make use of the 3D structure representations of molecules [31]. A pharmacophore is defined to be the molecular features pertinent to bioactivity aligned in three-dimensional spaces, including hydrogen bonding, charge transfer, and electrostatic and hydrophobic interactions [32]. The underlying methodology of pharmacophore model was defined by different researchers [33]. Recently, this model was successfully applied in mesangial cell proliferation inhibitor discovery and virtual screening of potential ligands for many targets such as HIV integrase and CCR5 antagonist [34–37]. In 3D pharmacophore model, the molecular spatial features and volume constraints represent the intrinsic interactions of small bioactive ligands with protein receptors. Wolber and Langer tried to extract ligand pharmacophores from protein cavities based on a defined set of six types of chemical structures [38] and develop the algorithms for ligand extraction and interpretation as well as pharmacophore creation for multiple targets.

Pharmacophore screening only considers those compounds who are direct mimics of the ligand from which the pharmacophore has been generated and may neglect the other positive binding modes as well. In fact, the pharmacophore model is limited to only one mode of action for small molecules [39]. However, this limitation can be conquered by combining multiple pharmacophore models with different modes of action. This method is called virtual parallel screening and has been successfully applied to the identification of natural products' activity [39, 40]. In such work, The PDB-based pharmacophores were firstly used for target fishing for TCM constituents. Results showed that 16 constituents of *Ruta graveolens* were screened against a database of pharmacophores, and good congruity was found between the potential predictions and their corresponding IC_50_ values.

Quantitative structure-activity relationships (QSARs) were first established in the early 1960s when computational means were used to quantitatively describe pharmacodynamics and pharmacokinetic effects in biology systems and the chemical structures of compounds [41]. Generally speaking, any mathematical model or statistical method that builds relationship between molecular structures and biological properties may be considered as QSAR. The idea of QSAR is easy while training and application of QSAR is much difficult since similar structures may interact with totally different targets due to the diversity and complexity of biology [42]. Furthermore, the intrinsic noise in data to describe both the chemical space and biological effects brings much trouble in accurate modeling [43]. Despite these difficulties, in case robust biological data is available and few outliers coexist, thousands of QSAR models have been generated and stored in related database in the past 40 years [44, 45].

### 3.2. Target-Based Approach

The target-based approach predicts ligand-target interactions by the structural information of protein targets as well as ligands. The target-based approach depends highly on the availability of the structural information of targets, either from wet experiments or numerical simulations [46, 47]. On one hand, these methods aim to predict the conformation and orientation of the ligand within the protein cavity. On the other hand, the binding affinity of the ligand and protein is simulated with scoring functions. The main target-based approach is docking, which predicts the preferred orientation of one molecule to another when they bind to each other to form a stable complex [48]. Usually, docking is implemented to search appropriate ligands for known targets with the lowest fitting energy. On the contrary, inverse docking seeks to fish targets from known ligands “from scratch” and also plays an important role in virtual screening.

Despite more than 20 years of research, docking and scoring ligands with proteins are still challenging processes and the performance is highly dependent on targets [49–51]. Docking cannot be applied to proteins whose 3D structures are not identified [52]. The high-resolution structure of the protein target is preferably obtained from X-ray crystallography and NMR spectroscopy. However, approximately half of the currently approved drugs bind to the membrane proteins, whose structures are extremely difficult to be acquired experimentally. Alternatively, homology modeling is usually adopted to build a putative geometry and docking cavity [53]. Besides, threading and ab initio structure prediction together with molecular dynamics (MD) and Monte Carlo simulations are utilized to predict the target structures. However, the fidelity of homology modeling, threading, and ab initio structures is still questioned by many researchers. Other important challenges of docking are the dynamic behavior, the large number of degrees of freedom, and the complexity of the potential energy surface. This confines docking to be a low-throughput method on a very small scale, which fails to predict interactions on the level of millions of ligands and targets. 

To alleviate the situation that docking depends on the nature of targets, multiple active sites have been used to compensate the ligand-dependent biases, and the consensus scoring has been also suggested to reduce the false positives in virtual screening [54]. The accuracy of scoring functions still remains the main weakness of docking approach [55]. Also, docking is starting to adopt the conformation information derived from protein-bound ligands as a strategy to overcome the limitations of current scoring functions and can predict the orientation of the ligands into the protein cavity [56]. Besides, molecular-dynamics-assisted docking method has been applied in virtual screening against the individual targets in HIV to search for multitarget drug-like agents, and KNI-765 was identified to be potential inhibitor [57].

Regardless of all limitations, virtual screening based on docking and inverse docking has been successfully utilized to identify and predict novel bioactive compounds in the past 10 years. Using the combinatorial small molecule growth algorithm, Grzybowski applied the docking to the design of picomolar ligands for the human carbonic anhydrase II [58, 59]. Inverse docking was firstly developed to identify multiple proteins to which a small molecule can bind or weakly bind. In some cases, the bioactivity of the TCM compounds is well recognized, while the underlying mode of action is not very clear. In 2001, INVDOCK [60] has been developed to search for the targets for TCM constituents and employed a database of protein cavities derived from PDB entries. The results of inverse docking involving multiple-conformer shape-matching alignment showed that 50% of the computer-predicted potential protein targets were implicated or experimentally validated. The same approach was used to determine potential drug toxicity and side effects in early stages of drug development, and results showed that 83% of the experimentally known toxicity and side effects were predicted [61]. Zahler et al. tried the inverse docking method to find potential kinase targets for three Indirubin derivatives and examined 84 unique protein kinases in total [62]. Recently, one indirubin compound was found to possess therapeutic effects against myelogenous leukemia [63].

Docking is usually used as the second step to further validate the ligand-target binding features after the first round of virtual screening by other ligand-based approaches [64–67]. Wei et al. applied the docking together with similarity search and molecular simulation to search for anti-SAS drugs [68], find the binding mechanism of H5N1 influenza virus with ligands [69], detect possible drug leading to Alzheimer's disease [70, 71], and identify the binding sites for several novel amide derivatives in the nicotinic acetylcholine receptors (AChRs) [72].

### 3.3. Machine Learning

The ligand-based approach and target-based approach predict potential ligand-target bindings by means of chemical similarity and structural information. Machine learning is a high-throughput method of artificial intelligence that enables computers to learn from data of knowns, including ligand chemistry, structural information, and ligand-protein networks, and to predict unknowns, such as new drugs, targets, and drug-target pairs. This method gains stability and credibility and has strong ability for classifications among large numbers of ligand-protein pairs that otherwise would be impossible to be connected based on chemical similarity alone. 

Machine learning is to exact features from data automatically by computers [73]. Basically, machine learning can be categorized into unsupervised learning and supervised learning. In unsupervised learning, the objective is to extract and conjecture patterns and interactions among a series of input variables, and there is no outcome to train the input variables. The common approaches in unsupervised learning are clustering, data compression, and outlier detection, such as principal-component-based methods [74]. In supervised learning, the objective is to predict the value of an outcome variable based on the input variables [75]. The data is commonly divided into training and validation datasets, which are used in turn to finalize a robust model. The variable the supervised model predicts is typically the binding probability of ligands and targets.

Nidhi et al. trained a multiple-category Laplacian-modified naïve Bayesian model from 964 target classes in WOMBAT and predicted the top three potential targets for compounds in MDDR with or without known targets information [76]. On average, the prediction accuracy with compounds with known targets is 77%. Bayesian classifier was usually used in early prediction, while the Winnow algorithm was reported more recently [77]. With the same training datasets, the prediction result is slightly different with the multiple-category Laplacian model. This indicates that it is necessary to apply different prediction methods and make comparisons even on the same training dataset.

The Gaussian interaction profile kernels, which represented the drug-target interactions, were used in regularized least squares combined with chemical and genomic space to achieve the prediction with precision-recall curve (AUPR) up to 92.7 [78]. Based on simple physicochemical properties extracted from protein sequences, the potential drug targets were related to the existing ones by several models [79]. The supervised bipartite graph inference is used to represent the drug interaction networks and can be solely able to predict new interactions, or together with chemical and genomic space [80, 81]. Besides, semisupervised learning method (Laplacian regularized least square FLapRLS) was also explored to effectively predict the results by integration of genomic and chemical space [82]. 

The support vector machine (SVM) is a powerful classification tool in which appropriate kernel functions are selected to map the data space into higher-dimensional space without increasing the computational difficulties. The performance of SVM is usually stronger than other probability-based models. Wale and Karypis [83] made comparisons between a Bayes classifier together with binary SVM, cascaded SVM, a ranking-based SVM, ranking perception, and the combination of SVM and ranking perception in terms of the ability to predict the targets for small compounds and found that the cascaded SVM has better performance than the Bayes models and thet the combination of SVM and ranking perception has the best performance of all. Kuhn et al. developed an SVM model based on the chemical-protein interactions from STITCH [84] using new features from ligand chemical space and interaction networks. Four new D-amino acid oxidase inhibitors were successfully predicted by this model and validated by wet experiments, and one may have a new application in therapy of psychiatric disorders other than being an antineoplastic agent [85].

Random forest, a form of multiple decision trees, recently has been applied to screen TCM database for potential inhibitors against several therapeutically important targets [86]. With the use of binding information from another database, random forest was performed to find multiple hits out of 8264 compounds in 240 Chinese herbs on an unbalanced dataset. Among all the predictions, 83 herb-target predictions were proved by the literature search. Three Potential inhibitors of the human, aromatase enzyme (CYP19) myricetin, liquiritigenin, and gossypetin, were screened by random forest as well as molecular docking studies. The virtual screening results were subsequently confirmed experimentally by *in vitro* assay [87]. 

Linear regression models have also been applied to predict ligand-target pairs. Zhao and Li developed a computational framework, drugCIPHER, to infer drug-target interactions based on pharmacology and genomic space [88]. In this framework, three linear regression models were created to relate drug therapeutic similarity, chemical similarity, and target similarity on the basis of a protein-protein interaction network. The drugCIPHER achieved the performance with AUC of 0.988 in the training set and 0.935 in the test set, and 501 new drug-target interactions were found, implying potential novel applications or side effects. 

Although machine learning has strong performance in classification of protein-ligand interactions, its shortcoming is obvious. The process of some machine learning methods is implicit, like a black box, from which we cannot have an intuitive biological or physical relevance between proteins and ligands. SVM maps the classification problem into higher space and acquires excellent performance with high computational efficiency. The tradeoff is that it can hardly explicitly create relationship between a protein and a ligand. Therefore, even with a very strong prediction tool, we can hardly move forward with innovations in theory of protein-ligand interactions.

## 4. Applications of Ligand-Protein Networks in TCM Pharmacology

Network-based pharmacology explores the possibility to develop a systematic and holistic understanding of the mode of actions of multidrugs by considering their multitargets in the context of molecular networks. It has also been suggested that relatively weak patterns of inhibition of many targets may prove more satisfactory than the highly potent single-target inhibitors routinely developed in the course of a drug discovery program [89]. In drug discovery, the use of networks incorporating multiple components and the corresponding multiple target, is one of the driving forces to propel the current development in TCM pharmacology. Several successful examples have been accumulated both in experiments and *in silico* analysis, as shown in Table 2.

### 4.1. Experimental Study

Many bioactive compounds in TCM herbs may have synergetic effort with many non-TCM drugs in markets. Tannin, a component derived from a TCM, can be combined with HIV triple cocktail therapy to yield everlasting efforts in preventing HIV virus propagation. The underlying mechanism is that tannin suppresses the activity of HIV-1 reverse transcriptase, protease, and integrase and cuts off virus fusion and virus entry into the host cells [90]. Recently, Li et al. proposed a new idea to induce immune tolerance in T cells by using matrine, a chemical derived from the root of *Sophora flavescens* AIT, targeting both the PKCy pathway and the NFAT pathway in cocktail preparations for treating AIDS [91].

Lam et al. recently showed in murine colon 38 allograft model that a formula containing 4 herbs (PHY906) has synergetic effect on reducing side effects and enhancing efficacy induced by CPT-11, a powerful anticancer agent with strong toxicity. The reason is that PHY906 can repair the intestinal epithelium by facilitating the intestinal progenitor or stem cells and several Wnt signaling components and suppressing a batch of inflammatory responses like factor kB, cyclooxygenase-2, and inducible nitric oxide synthase [92]. 

Multicomponent and multitarget interactions are the main mode of action for TCM formula, which exerts synergetic effects as a whole preparation rather than the primary active compound in TCM alone. Xie et al. demonstrated that other components in “Qingfu Guanjieshu” (QFGJS) could effectively influence the pharmacokinetic behavior and metabolic profile of paeonol in rats, indicating the synergy of herbal components. This synergy may be the result of enhanced adsorption of paeonol in the gastrointestinal tract induced by P-glycoprotein-mediated efflux change [93]. Another similar study showed that paeoniflorin from the root of *Paeonia lactiflora *was markedly enhanced when coadministrated with sinomenine, the stem of *Sinomenium acutum*. Sinomenine promotes intestinal transportation via inhibition of P-glycoprotein and affects the hydrolysis of paeoniflorin via interaction with b-glycosidase [94]. 

Huang-Lian-Jie-Du-Tang (HLJDT) is a TCM formula with anti-inflammatory efficacy, but the action mechanism is still not very clear. Zeng et al. investigated the effects of its component herbs and pure components on eicosanoid generation and found out the active components and their precise targets on arachidonic acid (AA) cascade. Results showed that *Rhizoma coptidis* and *Radix scutellariae* were the key herbs responsible for the suppressive effect of HLJDT on eicosanoid generation. Further experiments on the pure components of HLJDT revealed that baicalein derived from *Radix scutellariae* has significant inhibitory effect on 5-LO and 15-LO while coptisine from *Rhizoma coptidis* shows medium inhibitory effects on LTA(4)H. Besides, baicalein and coptisine were proven to have synergetic inhibition on LTB(4) by the rat peritoneal macrophages [95].

A TCM formula, Realgar-Indigo naturalis formula (RIF), was applied to treat Acute promyelocytic leukemia (APL) and showed a high complete remission (CR rate) [96]. In RIF, multiple agents within one formula were found to work synergistically. A small-scale combinational study using Chou and Talalay combination index method was performed and three main active components of RIF and six core proteins they targets in mediating the auti-tumor effect were identified. The main active ingredients of RIF are tetraarsenic tetrasulfide (A), indirubin (I), and tanshinone IIA (T), from Realgar, *Indigo naturalis*, and *Salvia miltiorrhiza,* respectively. A acts as the principal component of the formula, whereas T and I serve as adjuvant ingredients. ATI leads to ubiquitination/degradation of promyelocytic leukemia (PML) retinoic acid receptor oncoprotein, reprogramming of myeloid differentiation regulators, and G1/G0 arrest in APL cells by mediating multiple targets. Using multiomics technologies, Zhang et al. later proved that the combined use of Imatinib and arsenic sulfide from toxic herbal remedy exerted better therapeutic effects in a BCR/ABL-positive mouse model of chronic myeloid leukemia (CML) than either drug as a single agent. AS targets BCR/ABL through the ubiquitination of key lysine residues, leading to its proteasomal degradation, whereas IM inhibits the PI3 K/AKT/mTOR pathway [97].

### 4.2. Computational Framework

To target the complex, multifactorial diseases more effectively, the network biology incorporating ligand-protein networks has been applied in multitarget drug development as well as modernization of traditional Chinese medicine in the systematic and holistic way. Zhao et al. reviewed the available disease-associated networks, drug-associated networks that can be used to assist the drug discovery and elaborate the network-based TCM pharmacology [106]. Klipp et al. discussed the possibility to use networks to aid the drug discovery process and focused on networks and pathways in which the components are related by physical interactions or biochemical process [108]. Leung investigated the possibility of network-based intervention for curing system diseases by means of network-based computational models and using medicinal herbs to develop into new wave of network-based multitarget drugs. It was concluded that further integration across various “omics” platform and computational tools would accelerate the drug discovery based on network [109].

Barlow et al. screened among Chinese herbs for compounds that may be active against 4 targets in inflammation, by means of pharmacophore-assisted docking. The results showed that the twelve examples of compounds from CHCD inhibit multiple targets including cyclooxygenases 1 and 2 (COX), p38 MAP kinase (p38), c-Jun terminal-NH(2) kinase (JNK), and type 4 cAMP-specific phosphodiesterase (PDE4). The distribution of herbs containing the predicted active inhibitors was studied in regard to 192 Chinese formulae, and it was found that these herbs were in the formulae that were traditionally used to treat fever, headache, and so on [98]. 

Many traditional Chinese medicines (TCMs) are effective to relieve complicated diseases such as type II diabetes mellitus (T2DM). Gu et al. employed the molecular docking and network analysis to elucidate the action mechanism of a medical composition-Tangminling Pills which had clinical efficacy for T2DM. It was found that multiple active components in Tangminling Pills interact with multiple targets in the biological network of T2DM. The 37 targets were classified into 3 clusters, and proteins in each cluster were highly relevant to each other. Ten known compounds were selected according to their network attribute ranking in drug-target and drug-drug network [99].

XFZYD, a recipe derived from Wang Q. R. in Qing dynasty, was widely used in cardiac system disease. From similarity search and alignment, the chemical space of compounds in XFZYD was found to share a lot of similarities with that of drug/drug-like ligands set collected from cardiovascular pharmacology, while the chemical pattern in XFZYD is more diverse than that in drug/drug-like ligands for cardiovascular pharmacology. Docking protocol between compounds in XFZYD and targets related to cardiac system disease using LigandFit shows that many molecules have good binding affinity with the targeting enzymes and most have interactions with more than one single target. The active components in XFZYD mainly target rennin, ACE, and ACE2 in renin-angiotensin system (RAS), which modulates the cardiovascular physiological function. It was proved that promiscuous drugs in TCM might be more effective for treating cardiosystem diseases, which tend to result from multitarget abnormalities, but not from a single defect [100].

A lot of integrative computational tools and models have been developed and widely used to optimize the combination regimen of multicomponents drugs and elucidate the interactive mechanism among ligand-target networks. 

Li et al. built a method called distance-based mutual information model (DMIM) to identify useful relationships among herbs in numerous herbal formulae. DMIM combines mutual information entropy and distance between herbs to score herb interactions and construct herb network. Novel antiangiogenic herbs, Vitexicarpin and Timosaponin A-III, were discovered to have synergistic effects. Based on herb network constructed by DMIM from 3865 collateral-related herbs, the interactions between TCM drugs and disease genes in cancer pathways and neuro-endocrine-immune pathways were inferred to contribute to the action of Liu-wei-di-huang formula, one of the most well-known TCM formulae as potential treatment for a variety of diseases including cancer, dysfunction of the neuro-endocrine-immune-metabolism system, and cardiovascular system [101].

Wang et al. adopted a new method based upon lattice experimental design and multivariate regression to model the quantitative composition-activity relationship (QCAR) of *Shenmai*, a Chinese medicinal formula. This new strategy for multicomponent drug design was then successfully applied in searching optimal combination of three key components (PD, PT, and OP) of *Shenmai*. Experimental outcome of infarct rate of heart in mice with different dosage combination of the three components was finally measured, and the fitted relationship equation showed that the optimal values of PD, PT, and OP were 21.6, 39.2, and 39.2%, respectively [102]. The proportion of two active components of Qi-Xue-Bing-Zhi-Fang, PF and FP, was also optimized in similar way using several fitting techniques like linear regression, artificial neural network, and support vector regression [103]. Although the underlying mechanism of drug synergy for the two formulae was still not very clear, the interactions of multiple weak bindings among different compounds and targets might be the contributory factors. 

A network-based multitarget computational scheme for the whole efficacy of a compound in a complex disease was developed for screening the anticoagulant activities of a serial of argatroban intermediates and eight natural products, respectively. Aimed at the phenotypic data of drugs, this scheme predicted bioactive compounds by integrating biological network efficiency analysis with multitarget docking score, which evolves from the traditional virtual screening method that usually predicted binding affinity between single drug molecule and target. A ligand can have impact on multiple targets based on the docking scores, and those with highest-target-network efficiency are regarded as potential anticoagulant agents. Factor Xa and thrombin are two critical targets for anticoagulant compounds, and the catalytic reactions they mediate were recognized as the most fragile biological matters in the human clotting cascade system [104].

Sun et al. presented a systematic target network analysis framework to explore the mode of action of anti-Alzheimer's disease (AD) herb ingredients based on applicable bioinformatics resources and methodologies on clinical anti-AD herbs and their corresponding target proteins [105]. The results showed that, just as many FDA-approved anti-AD drugs do, the compounds of these herbs bind to targets in AD symptoms-associated pathway. Besides, they also interact closely with many successful therapeutic targets related to diseases such as inflammation, cancer, and diameters. This suggests that the possible cross talks between these complicated diseases are prevalent in TCM anti-AD herbs [110]. Moreover, pathways of Ca(2+) equilibrium maintaining, upstream of cell proliferation and inflammation, were found to be intensively hit by the anti-AD herbal ingredients.

Based on the available experimental results, Zhao et al. analyzed the molecular mechanism with the aid of pathways and networks and theoretically proved the multitarget effect of St. John's Wort [106]. A comprehensive literature search was conducted and the neurotransmitter receptors, transporter proteins, and ion channels on which the SJW active compounds show effects were collected. Three main pathways that SJW intervenes were found by mapping these proteins onto KEGG pathways. Active components in SJW mainly intervene with neuroactive ligand-receptor interaction, the calcium-signaling pathway, and the gap-junction-related pathway, pertinent to targets including NMDA-receptor, CRF1 receptor, 5-hydroxytryptamine receptor 1D, and dopamine receptor D1. The networks show that the effect of SJW is similar to that of combinations of different types of antidepressants. However, the inhibitory effects of the SJW on each of the pathway are lower than other individual agents. Accordingly, the significant antidepressant efficacy and lower side effects are due to the synergetic effect of low-dose multitarget actions. 

Zhang et al. established an integrative platform of TCM network pharmacology to discover herbal formulae on basis of systematic network. This platform incorporates a set of state-of-the-art network-based methods to explore the action mechanism, identify active ingredients, and create new synergetic combinations of components. The Qing-Luo-Yin (QLY), an antirheumatoid arthritis (RA) formula, was studied comprehensively using the new platform. It is found that the target network of QLY is involved in RA-related key processes including angiogenesis, inflammatory response, and immune response. The four herbs in QLY work in concert to promote efficiency and reduce toxicity, as the *jun*, *chen*, *zuo*, and s*hi* in Chinese, respectively. Specifically, the synergetic effect of Ku-Shen (*jun* herb) and Qing-Feng-Teng (*chen* herb) may come from the feedback loop and compensatory mechanisms [107].

## 5. Discussion and Conclusion

In recent years, the bottleneck in western medicine has brought unprecedented opportunities in TCM research and development. For decades, the fundamental research has achieved great success and laid the foundation of modern western medicine, and the philosophical idea of “reductionism” was considered to own the credit.

The counterparty of “reductionism” in Chinese medicine is the philosophical idea of holism, which has thousands years of history of practice in China as well as in other Asian countries. Using this methodology, the effectiveness of TCM can only be verified from a large number of clinical trials given the unclear composition and unknown relationship among various components. This implicit effect without clear clarification at the molecular level has been hindering the modernization of TCM. How to learn from the accumulative knowledge of western medicine in order to identify the effective compositions and explore the molecular mechanism of the efficacy is an urgent problem that needs to be solved in TCM.

**Figure 1:**
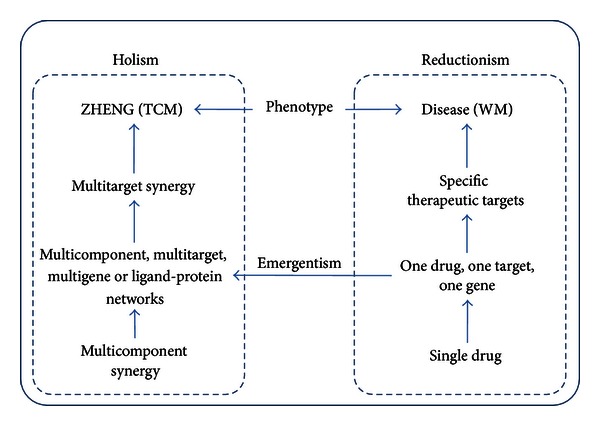
Unity of opposites on holism in traditional Chinese medicine and reductionism in western medicine. Emergentism constructs the framework of the understanding of holism in TCM via accumulative practice of reductionism in WM.

The hypothesis of “multidrug, multitarget, multigene” in fact bridges the gap between TCM and western medicine and is also a manifestation of unity of opposites on “reductionism” and “holism.” TCM uses the holistic method to investigate the effects of multicomponent formula across the whole organism, such as the use of a variety of “ZHENG” in TCM theory [111]. However, the only option we have to uncover the underlying mechanism of TCM at the molecular level is to make use of the theory of reductionism. Of course, for complex systems, the reduction method can only reach to a certain depth since it becomes more troublesome as we get deeper. Therefore, some researchers tend to reduce the mechanism of TCM to the level of “multidrug, multitarget, multigene” at present, and for further reduction to the level of “single-drug, single-target, single-gene,” the problem of emergentism [112] in philosophy needs to be addressed properly. The theory of emergentism believes that some unique features or “ultimate features” of a system can never be reduced to properties at lower levels, nor the former can be predicted or explained by the latter, as shown in Figure 1.

So far, ligand-protein network or “multidrug, multitarget, multigene” is one of the few basic modules that can clearly reveal the pharmacology of TCM and is expected to be the future direction of the modernization of TCM. But just relying on experimental scientists to build ligand-protein interactions nonexhaustively will slow down both the modernization of TCM and the development of its industry. Therefore, the use of cross-platform database (TCM compounds and recipe database; see Section 2 in this paper) and the improvement on modeling technique (computational method of ligand-protein interactions; see Section 3 in this paper) will afford the basis of *in silico* research for future modernization and development of TCM. It can be foreseen that one future direction is to use these TCM databases and predictive models to reveal the pharmacological effect of TCM, through the establishment of ligand-protein networks or, “multidrug, multitarget, multigene” relationships. Nevertheless, the pharmacological mechanism of TCM can be very complex and may not be well explained only with the known ligand-protein network. After all, this is a process of reeling silk from cocoons and also one of the best choices we have right now.

The increasing availability of ligand-protein networks is a unique chance to boost success in the modernization of TCM based on the accumulative knowledge of TCM formulae and practices based on the assumption that TCM exerts the pharmacological efficacy in multidrug, multitarget way. Although preliminary research has been initiated in this area, there is still a long way to go to further leverage these networks and modeling techniques. Virtual screening and informatics in the drug discovery area have already been proven to be quite useful either to predict potential new drug and target candidates for experimentalists or to explore the functional mechanism at the molecular level. A large number of drug-target interactions have thus been gained and the resulted drug-target networks will also be quite beneficial to investigate the underlying mechanism of multicomponent drugs, such as the TCM. With further applications of these methods in TCM area, we are expecting to reveal the mode of action underlying polypharmacology of TCM. This grants us the possibility to discover novel effective drug leads, understand the synergistic mechanism of drug combinations, and more importantly, develop drug portfolios against epidemic, chronic disease, cancer, and other complex diseases that are almost incurable by western medicine. 

## Figures and Tables

**Table 1:** Basic information for main TCM databases.

Database	Description	URL or ref.
Traditional Chinese medicine database (TCMD)	6760 herbs, 23,033 compounds	[12]
Chinese herb constituents database (CHCD)	240 herbs, 8264 compounds	[15]
3D structural database of biochemical components	2073 herbs, 10,564 compounds	[16]
TCM database@Taiwan	453 herbs, 20,000 compounds	[17]
Traditional Chinese medicine information database (TCM-ID)	1197 formulae, 1313 herbs, ~9000 compounds	[18]
TCM drugs information system	1712 formulae, 2738 herbs, 16,500 compounds, 868 dietotherapy prescription	[19]
Comprehensive herbal medicine information system for cancer (CHMIS-C)	203 formulae, 900 herbs, 8500 compounds	[20]
China natural products database (CNPD)	45,055 compounds	[21]
Marine natural products database (MNPD)	8078 compounds, 3200 with bioactivity data	[22]
Bioactive plant compounds database (BPCD)	2794 compounds	[15]
acupuncture.com.au	TCM formulations	http://www.acupuncture.com.au/education/herbs/herbs.html/
Dictionary of Chinese herbs	TCM formulae, toxicity, and side effects	http://alternativehealing.org/chinese_herbs_dictionary.htm/
Plants for a future	Herb medical usage and potential side effects	http://www.pfaf.org/

**Table 2:** Summary of multi-target drugs/preparations with TCM pharmacology based on ligand-protein networks.

Disease	Methods and experiments	Formula, herbs, and components	TCM pharmacology	Reference
AIDS	Experiments	Tannin	Tannin suppresses the activity of HIV-1 reverse transcriptase, protease, and integrase and cuts off virus fusion and virus entry into the host cells.	[90]

AIDS	Experiments	Matrine from the root of *Sophora flavescens *	Matrine is effective in inducing T-cell anergy by targeting both the MAPKs pathway and the NFAT pathway.	[91]

Antitumor	Experiments	PHY906: *Glycyrrhiza uralensis* Fisch (G), *Paeonia lactiflora* Pall (P), *Scutelleria baicalensis* Georgi (S), and *Ziziphus jujuba* Mill (Z).	PHY906 reduces CPT-11-induced gastrointestinal toxicity in the treatment of colon or rectal cancer by several mechanisms. It both repairs the intestinal epithelium by facilitating the generation of intestinal progenitor or stem cells and several Wnt signaling components and suppresses inflammatory responses like factor kB, cyclooxygenase-2, and inducible nitric oxide synthase.	[92]

Anti-inflammatory and analgesic effects	Experiments	Qingfu Guanjieshu (QFGJS): paeonol and other components	The pharmacokinetic behavior and metabolites of paeonol are greatly promoted by other components in QFGJS. This may be the result of enhanced adsorption of paeonol in the gastrointestinal tract by P-glycoprotein-mediated efflux change.	[93]

Inflammatory and arthritic diseases	Experiments	Paeoniflorin from the root of *Paeonia lactiflora* and sinomenine from the stem of *Sinomenium acutum*.	Paeoniflorin is markedly enhanced when coadministrated with sinomenine, which promotes intestinal transportation via the inhibition of P-glycoprotein and affects the hydrolysis of paeoniflorin via interaction with b-glycosidase.	[94]

Anti-inflammatory	Experiments	Huang-Lian-Jie-Du-Tang (HLJDT): *Rhizoma coptidis* and *Radix scutellariae *	Baicalein derived from Radix scutellariae showed significant inhibitory effect on 5-LO and 15-LO while coptisine from Rhizoma coptidis showed medium inhibitory effects on LTA(4)H.	[95]

Acute promyelocytic leukemia (APL)	Experiments	Realgar-Indigo naturalis: tetraarsenic tetrasulfide (A), indirubin (I), and tanshinone IIA (T)	ATI leads to ubiquitination/degradation of promyelocytic leukemia (PML) retinoic acid receptor oncoprotein, reprogramming of myeloid differentiation regulators, and G1/G0 arrest in APL cells by mediating multiple targets. A acts as the principal component of the formula, whereas T and I serve as adjuvant ingredients.	[96]

Chronic myeloid leukemia (CML)	Experiments	Imatinib (IM) and arsenic sulfide [As(4)S(4) (AS)]	AS targets BCR/ABL through the ubiquitination of key lysine residues, leading to its proteasomal degradation, whereas IM inhibits the PI3K/AKT/mTOR pathway.	[97]

Inflammation	Pharmacophore-assisted docking	Twelve examples of compounds from CHCD	The screened compounds target cyclooxygenases 1 and 2 (COX), p38 MAP kinase (p38), c-Jun terminal-NH(2) kinase (JNK), and type 4 cAMP-specific phosphodiesterase (PDE4).	[98]

Type II diabetes mellitus (T2DM)	Molecular docking (LigandFit), clustering, and drug-target network analysis	676 compounds in eleven herbs from Tang-min-ling Pills	Multiple active components in Tangminling Pills interact with multiple targets. The 37 targets were classified into 3 clusters, and proteins in each cluster were highly relevant to each other. Ten known compounds were selected according to their network attribute ranking in drug-target and drug-drug network.	[99]

Cardiovascular disease	Similarity search and alignment, docking (LigandFit)	Xuefu Zhuyu decoction (XFZYD): 501 compounds, 489 drug/drug-like compounds	Active components in XFZYD mainly target rennin, ACE, and ACE2 in renin-angiotensin system (RAS), which modulates the cardiovascular physiological function.	[100]

9 types of cancer, 5 diseases with dysfunction, and 2 cardiovascular disorders	Distance-based mutual information model (DMIM)	Liu-wei-di-huang formula (LWDH), Shan-zhu-yu (*Fructus Corni*), Ze-xie (*Rhizoma Alismatis*), Dan-pi (*Cortex Moutan*), Di-huang (*Radix Rehmaniae*), Fu-ling (*Poria Cocos*) and Shan-yao (*Rhizoma Dioscoreae*)	The interactions between TCM drugs and disease genes in cancer pathways and neuro-endocrine-immune pathways were inferred to contribute to the action of LWDH formula.	[101]

Cardiovascular diseases	Quantitative composition-activity relationship model (QCAR) (SVM and linear regression)	*Shenmai*, Qi-Xue-Bing-Zhi-Fang (QXBZF)	The proportion of active components of *Shenmai* and QXBZF were optimized based on clinical outcome (collateral and infarct rate of heart) using QCAR. The interactions of multiple weak bindings among different compounds and targets may contribute to the synergetic effect of multicomponent drugs.	[102, 103]

Anticoagulant	Network-based computational scheme utilizing multi-target docking score (LigandFit and AutoDock)	Six argatroban intermediates and a series of components from 24 TCMs widely used for cardiac system diseases	A ligand can have impact on multiple targets based on the docking scores, and those with the highest-target network efficiency are regarded as potential anticoagulant agents. Factor Xa and thrombin are two critical targets for anticoagulant compounds and the catalytic reactions they mediate were recognized as the most fragile biological matters in the human clotting cascade system.	[104]

Alzheimers' disease	Systematical target network analysis framework	*Ginkgo biloba*, *Huperzia serrata*, *Melissa officinalis*, and *Salvia officinalis *	AD-symptoms-associated pathways, inflammation-associated pathways, cancer-associated pathways, diabetes-mellitus-associated pathways, Ca2*þ*-associated pathways, and cell-proliferation pathways are densely targeted by herbal ingredients.	[105]

Depression	Literature search and network analysis	Hyperforin (HP), hypericin (HY), pseudohypericin (PH), amentoflavone (AF), and several flavonoids (FL) from St. John's Wort (SJW)	Active components in SJW mainly intervene with neuroactive ligand-receptor interaction, the calcium-signaling pathway, and the gap-junction related pathway. Pertinent targets include NMDA-receptor, CRF1 receptor, 5-hydroxytryptamine receptor 1D, and dopamine receptor D1.	[106]

Rheumatoid arthritis (RA)	Integrative platform of TCM network pharmacology including drugCIPHER	Qing-Luo-Yin (QLY), including four herbs, Ku-Shen (*Sophora flavescens*), Qing-Feng-Teng (*Sinomenium acutum*), Huang-Bai (*Phellodendron chinensis*) and Bi-Xie (*Dioscorea collettii*), which contain several groups of ingredients such as saponins and alkaloids	The target network of QLY is involved in RA-related key processes including angiogenesis, inflammatory response, and immune response. The four herbs in QLY work in concert to promote efficiency and reduce toxicity. Specifically, the synergetic effect of Ku-Shen (*jun* herb) and Qing-Feng-Teng (*chen* herb) may come from the feedback loop and compensatory mechanisms.	[107]
